# Prehabilitation before pancreatoduodenectomy: results of a retrospective single-center study

**DOI:** 10.1186/s13741-026-00685-2

**Published:** 2026-04-23

**Authors:** Philipp R. Scherber, Jana Bauer, Sebastian Holländer, Gereon Gäbelein, Evelyn Marth, Laura Brusokas, Samra Gafarli, Peter Jacob, Matthias Glanemann

**Affiliations:** Department for General Surgery, Visceral-, Vascular- and Paediatric Surgery, Saarland University Medical Center, Homburg, 6642 Germany

**Keywords:** Pancreatectomy, Perioperative care, Postoperative complications, Surgery

## Abstract

**Background:**

Major surgeries like pancreatoduodenectomy carry high risks for perioperative complications, especially in older patients and those with comorbidities. While perioperative care has improved, the role of prehabilitation—defined as structured preoperative physical and nutritional preparation—remains underexplored in pancreatic surgery. This study evaluated the impact of a multimodal prehabilitation program on postoperative outcomes in patients undergoing pancreatoduodenectomy.

**Methods:**

A retrospective single-center matched-pair analysis was conducted including patients who underwent elective pancreatoduodenectomy after implementation of a structured prehabilitation program (January 2023-October 2024) and a historical control group. Matching criteria were sex, ASA class, and type of procedure. The multimodal home-based prehabilitation program comprised physical exercise, breathing training, and individualized nutritional support. Primary outcomes were postoperative complications, length of hospital stay, and recovery parameters. Multivariable logistic regression and propensity score-based inverse probability of treatment weighting (IPTW) analyses were performed to adjust for confounding.

**Results:**

Of 116 eligible patients, 36 completed prehabilitation and were matched to 36 controls. Overall postoperative complications were lower after prehabilitation (44.4% vs. 66.7%, *p* = 0.053), while severe complications (Clavien-Dindo 3–4) were significantly reduced (16.7% vs. 38.9%, *p* = 0.042). Postoperative blood transfusion rates were lower (5.6% vs. 22.2%, *p* = 0.041), and hospital stay was shorter (13 vs. 16 days, *p* = 0.017). IPTW-adjusted analyses confirmed a consistent reduction in postoperative complications (OR 0.425), although statistical significance was not consistently reached. Subgroup analysis of patients ≥ 80 years demonstrated a significant reduction in complications (25% vs. 100%, *p* = 0.028).

**Conclusions:**

Prehabilitation is feasible and associated with improved postoperative outcomes after pancreatoduodenectomy, particularly regarding severe complications and length of hospital stay. Although limited by sample size and adherence, the findings suggest that prehabilitation may enhance recovery, especially in elderly patients. Larger prospective studies are warranted to confirm these results.

**Trial registration:**

The study was registered on June 18, 2025 in the German Clinical Trials Register (DRKS) under the identification number DRKS00037231.

## Background

Major surgeries, particularly visceral oncological procedures, are among the most important risk factors for perioperative morbidity and mortality (Dobson 2020; Shrikhande et al. 2013). Thanks to continuous improvements in peri- and postoperative management, including standardized clinical pathways and enhanced recovery after surgery (ERAS) programs, patient safety and quality of care have substantially improved over the past decades (Haynes et al. 2009; Téoule et al. 2019).

However, pancreatic surgery, and in particular pancreatoduodenectomy, remains associated with considerable postoperative morbidity rates of up to 50% and higher, even in high-volume centers (Theijse et al. 2023). This is mainly attributable to the extensive surgical trauma and the limited physiological reserve of many patients, who frequently present with advanced age, sarcopenia, malnutrition, and multiple comorbidities. Although perioperative management has improved substantially, complication rates remain high, especially among frail and elderly individuals (Baquero and Rich 2015). Strengthening patients’ physiological reserve prior to surgery therefore appears to be a promising strategy to mitigate surgical stress and enhance postoperative recovery (Bundred et al. 2020). Prehabilitation is a structured preoperative approach designed to enhance patients’ physical and functional capacity, thereby improving their ability to tolerate and recover from surgery (Fleurent-Grégoire et al. 2024). This multimodal concept typically combines physical exercise, nutritional optimization, and psychological support (Fleurent-Grégoire et al. 2024; Sliwinski et al. 2023). Prehabilitation has already been investigated in several oncological settings; patients with esophagogastric or colorectal cancer, for example, may benefit from prehabilitation during neoadjuvant therapy (Bausys et al. 2022; Molenaar et al. 2023). However, current evidence remains heterogeneous with respect to patient selection, intervention protocols, duration, and outcome measures, precluding meaningful comparisons and consistent recommendations regarding risk assessment, exercise regimens, and nutritional interventions (Sliwinski et al. 2023; Bausys et al. 2022). A recent systematic review published in Clinical Nutrition ESPEN further highlights the growing interest in prehabilitation for hepatopancreatobiliary surgery while emphasizing the variability of existing study designs and the need for procedure-specific data (Refaat et al. 2024).

In contrast to other gastrointestinal malignancies, neoadjuvant treatment strategies are less consistently implemented in primarily resectable pancreatic cancer. In these patients, prompt surgical resection remains the standard of care, limiting the available time window for structured preoperative interventions (Zhan et al. 2017; Strobel et al. 2019). Delaying surgery in favor of prolonged prehabilitation may entail the risk of tumor progression, which necessitates a careful balance between oncological urgency and potential functional optimization. Consequently, the feasibility, timing, and clinical impact of prehabilitation in patients undergoing pancreatoduodenectomy require further investigation.

Therefore, the aim of the present study was to evaluate the impact of a structured prehabilitation program on peri- and postoperative outcomes in patients undergoing pancreatoduodenectomy. Specifically, we sought to determine whether prehabilitation, consisting of physical exercise and nutritional support, can reduce postoperative complications, shorten hospital stay, and improve recovery.

## Methods

Prehabilitation before pancreatic surgery was introduced in the Department for General Surgery, Visceral-, Vascular- and Paediatric Surgery on January 1, 2023. All patients who were scheduled for elective pancreatoduodenectomy from January 1, 2023 until October 1, 2024 were included in this study.

First, adherence to the prehabilitation program and the dropout rate were analyzed. Subsequently, a retrospective, single-center, matched-pair study with a prospectively implemented prehabilitation intervention and a historical control group was conducted in which the peri- and postoperative outcomes of patients who underwent prehabilitation were compared with a consecutive series of patients that underwent pancreatoduodenectomy without prehabilitation. The matching criteria were sex, ASA class and the surgical procedure, classical Whipple or pylorus preserving Whipple procedure.

At the initial consultation, all patients who were scheduled for pancreatoduodenectomy received written and oral information about the prehabilitation program, its background and aims. Upon consent, the patients were provided with a logbook for information and documentation purposes, containing detailed information on supplementary nutrition as well as muscle strengthening and endurance training, and breathing exercises. Each exercise was designed to allow participants to perform it at home without the need for elaborate equipment. Participants were instructed to document each completed exercise in the logbook. The registration of basic demographic information (body weight and height, body mass index (BMI), nutritional risk score (NRS)) and the prescription of supplementary nutrition were carried out by the clinic´s dietitians and study nurses. Prehabilitation covered the period from the initial consultation at our center for hepato-pancreatico-biliary surgery until the day of surgery; surgery was not postponed for the purpose of prehabilitation. During the prehabilitation, participants were called at least once by a study nurse to ensure that the program was being followed as intended and to address any questions patients might have.

An interval of less than seven days from the initial consultation to surgery, mental or physical inability to participate as well as explicit refusal or inadequate adherence to the program, were considered exclusion criteria. Patients were classified as non-adherent if, during this minimum period of seven days, the prescribed exercises or nutritional therapy were not performed as intended on at least one of the seven days, or if they failed to follow the regimen on more than 14% of days during the entire individualized prehabilitation period.

The peri- and postoperative treatment and complication management following pancreatoduodenectomy are standardized in the Department for General Surgery, Visceral-, Vascular- and Paediatric Surgery according to clinical treatment pathways, ensuring no difference between patients receiving prehabilitation, those who did not complete the intervention and the control group. Furthermore, all surgeries were performed by the two surgeons that were designated for hepato-pancreatico-biliary surgeries, the chief physician and the senior physician of the department.

The pylorus preserving pancreatoduodenectomy was the preferred procedure; the classical pancreatoduodenectomy was only performed if there were indications of tumor adherence or infiltration into the pylorus or distal stomach (Glanemann et al. 2008a). A standard lymphadenectomy according to the recommendations of the International Study Group of Pancreatic Surgery (ISGPS) was performed in all cases (Tol et al. 2014). If tumor infiltration into the portal vein and/or superior mesenteric vein was suspected, concomitant resection and reconstruction by end-to-end anastomosis or interposition of an autologous venous graft was performed in order to achieve negative margins (Glanemann et al. 2008b). After removal of the specimen, pancreaticojejunostomy or pancreatogastrostomy, hepaticojejunostomy and a duodenojejunostomy, or gastrojejunostomy in case of the classical Whipple procedure, were performed in turn (Hirono et al. 2019; Bartsch et al. 2012).

The study was submitted to the Ethics Committee of the Medical Association of Saarland (Ärztekammer des Saarlandes) and received approval (reference number: 69/25, date 15/05/2025).

The study was registered with the German Clinical Trials Register under the identification number DRKS00037231.

### Data collection

Demographic data including age, sex, body weight and height, body mass index, duration of the prehabilitation period, patients´ physical status according to American Society of Anaesthesiologists Status System (ASA class), tumor markers CEA and CA 19–9, comorbidities, preoperative biliary drainage were recorded. Intraoperative data such as surgical procedure (classical Whipple or pylorus preserving Whipple procedure), type of pancreatic anastomosis (pancreatogastrostomy or pancreaticojejunostomy), intraoperative blood loss and blood transfusion rate were gathered. The severity of postoperative complications was classified according to the Clavien-Dindo classification system. Postoperative surgical complications, pancreatic fistula (POPF), bile leakage, postoperative haemorrhage, delayed gastric emptying (DGE) were defined according to the International Study Group of Pancreatic Surgery and the International Study Group of Liver Surgery (Koch et al. 2011; Bassi et al. 2017; Wente et al. 2007). Furthermore, wound healing impairments, fascial dehiscence, pulmonary or cardiac complications, as well as the length of hospital and intensive care unit (ICU) stay and the intrahospital mortality were recorded. The readmission rate was examined on day 30 and 90 after patients´ discharge from the hospital. All information was obtained from the hospital information system.

### Preoperative exercise and nutritional therapy

Patients who participated in the prehabilitation program were offered a daily exercise routine consisting of breathing training along with alternating strength or endurance exercises.

The breathing exercises consisted of the following elements:

- thoracic mobilization using lateral stretching and rotation (15 repetitions per side)
- abdominal and lateral breathing with arms raised overhead (15 times with two repetitions)
- breathing exercises with the "Triflow Atemtrainer” (5 repetitions, three times a day)
- Performing pursed-lip breathing: Inhaling through the nose and then exhaling against resistance with closed lips (five times with two repetitions).

Elements of muscle strengthening training:

- leg abduction at the hip joint (15 times with two repetitions)
- flexion and extension of the leg at the hip joint (15 times with two repetitions)
- standing on tiptoes and holding for ten seconds (five times with two repetitions)
- quickly getting up and sitting down on a chair (ten times with two repetitions)
- bicep and tricep exercises with light weights of one to two kilograms (ten times with three repetitions).

Elements of endurance training:

- squats (ten times with two repetitions)
- brisk walking (45 min)
- stair climbing 100 steps, if possible.

The physical activity was accompanied by an individualized nutrition plan. Depending on the BMI and NRS at the initial assessment, patients were recommended one or two high-caloric supplementary meals (Myketo, Swiss Medical Food): for a BMI < 20 kg/m^2^ and/or NRS < 3, two times per day, otherwise, once per day.

### Statistics

Continuous variables were tested for normal distribution using the Shapiro–Wilk test. Normally distributed data are presented as mean ± standard deviation (SD), whereas non-normally distributed data are reported as median with interquartile range (IQR). For comparisons between groups, Student’s t-test was used for normally distributed continuous variables. The Mann–Whitney U test was applied for non-normally distributed continuous variables. Categorical variables are presented as absolute numbers and percentages and were compared using the Chi-square test or Fisher’s exact test, as appropriate. Variables associated with postoperative complications at a significance level of *p* < 0.10 in univariate analysis were entered into a multivariate logistic regression model to identify independent predictors. Results of the multivariate analysis are reported as odds ratios (OR) with 95% confidence intervals (CI). In addition, a propensity score-based inverse probability of treatment weighting (IPTW) analysis was performed. The propensity score for receiving prehabilitation was estimated using logistic regression including the following preoperative covariates selected a priori: age, sex, ASA class, nutritional risk score (NRS), preoperative biliary drainage, pulmonary comorbidity, and coronary artery disease. Age was modeled as a continuous variable in the primary IPTW analysis. As a sensitivity analysis, age was additionally modeled as a categorical variable (< 60, 60–69, 70–79, and ≥ 80 years). Stabilized inverse probability weights for the average treatment effect were calculated and truncated at the 1st and 99th percentiles to limit the influence of extreme weights. Covariate balance before and after weighting was assessed using standardized mean differences (SMD), with an absolute SMD < 0.10 considered indicative of acceptable balance. Weighted outcome analyses were then performed using survey-weighted generalized linear models. For binary outcomes, weighted logistic regression models were fitted and results are reported as odds ratios (OR) with 95% confidence intervals (CI). Length of postoperative hospital stay was analyzed using a weighted linear model. As a robustness check, additional multivariable logistic regression models for postoperative complications were fitted with adjustment for treatment group, age, sex, ASA class, NRS, and preoperative biliary drainage. Because of the very low number of events, intrahospital mortality was reported descriptively and was not used as a primary modeled outcome. All statistical tests were two-sided, and a p-value ≤ 0.05 was considered statistically significant. Statistical analysis was performed using SPSS software, version 29 (SPSS Inc, Chicago, Ill).

## Results

**Fig. 1:**
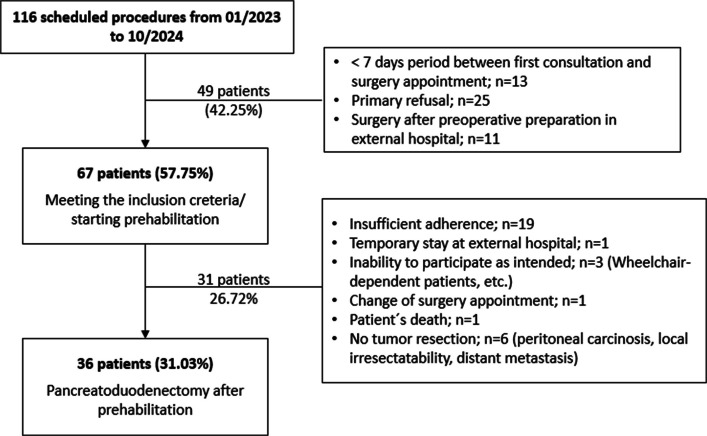
Patient selection

Within the 22 months interval from January 2023 to October 2024 116 patients were scheduled to undergo pancreatoduodenectomy. Out of these patients, 49 patients (42.25%) did not meet the inclusion criteria (Fig. 1) as they declined participation at the initial stage (*n* = 25), the interval until surgery was less than 7 days (*n* = 13), or they were transferred for surgery after receiving preoperative treatment at an external hospital (*n* = 11). The remaining 67 patients (57.75%) started prehabilitation. Due to insufficient adherence (*n* = 19), temporary stay at another hospital (*n* = 1), comorbidities that prevent patients from participation as intended (*n* = 3), change of surgery appointment (*n* = 1), unexpected death of one patient, or intraoperative irresectability of the tumor because of histopathologically proven peritoneal or distant metastases (n = 6), another 31 patients (26.72%) were excluded from the final analysis. Pre- and perioperative parameters as well as the postoperative outcome of the remaining 36 patients (31.03%) that underwent pancreatoduodenectomy after successfully completing prehabilitation were subsequently compared with a control group of 36 matched patients that underwent the same procedure in the last two years before. The matching criteria were sex, ASA class and the surgical procedure, the pylorus preserving or classical Whipple procedure.

**Fig. 2:**
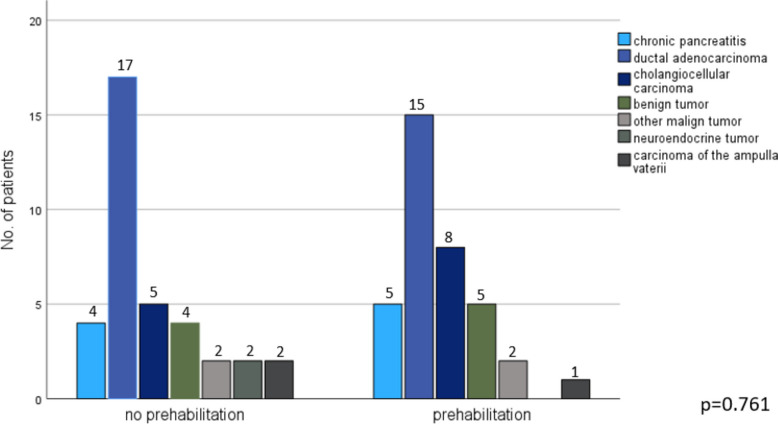
Histopathological findings

Regarding background factors of patients who underwent prehabilitation before surgery and those who did not, there were no significant differences, including demographic characteristics and comorbidities, except for the prevalence of a preoperative biliary drainage, which was higher in the prehabilitation group (Table 1). All patients underwent primary resection without neoadjuvant therapy. The median interval from initial consultation to surgery was 16 days (IQR 12–22 days) in the prehabilitation group and 15 days (IQR 8–35 days) in the control group, corresponding to a median difference of 1 day (*p* = 0.951). Furthermore, there were no statistically significant differences between the two groups comparing the indications for surgery with the ductal pancreatic adenocarcinoma being the predominant reason (Fig. 2). As shown in Table 1, the pylorus preserving Whipple procedure was the most commonly performed procedure in both groups. With 75% in the no prehabilitation group and 83.33% in the prehabilitation group, the pancreaticojejunostomy was the predominant kind of pancreatic anastomosis. The median operation time and estimated blood loss were nearly the same in both groups (Table 1). In the postoperative course, patients who underwent prehabilitation before surgery showed a lower rate of postoperative complications (44.44% vs. 66.67%, absolute difference −22.23%); however, this difference did not reach statistical significance (*p* = 0.053). Severe postoperative complications (Clavien-Dindo grade 3–4) were significantly reduced in the prehabilitation group (16.67% vs. 38.89%, difference −22.22%, *p* = 0.042). In addition, the need for postoperative blood transfusions was also significantly lower in these patients (5.56 vs. 22.22%, difference −16.66%, *p* = 0.041). The median stay on ICU was one day in both groups. The median hospital stay after surgery was significantly shorter in the prehabilitation group compared to the control group (13 vs. 16 days, difference −3 days, *p* = 0.017). Thirty- and ninety-day readmission rates were lower in the prehabilitation group, although not statistically significant (8.33% vs. 13.89%, difference −5.56%, *p* = 0.453 and 11.11% vs. 13.89%, difference −2.78%, *p* = 0.791, respectively) (Table 2).

**Table 1:** Basic demographic and intraoperative characteristics

	**no prehabilitation**	**prehabilitation**	***p*-value**
Age [Med (IQR)]	68 (61–75) y	68 (61–75) y	0.901
Sex (w/m)	13/23 (36.11%/63.89%)	13/23 (36.11%/63.89%)	1.000
BMI [Med (IQR)]	25 (22.50–27.50) kg/m^2^	23.30 (20.60–25.30) kg/m^2^	0.161
ASA (ASA 2/ASA 3)	25/11 (69.44%/30.56%)	25/11 (69.44%/30.56%)	1.000
Preoperative period* [Med (IQR)]	15 (8–35) d	16 (12–22) d	0.951
CA 19–9 [Med (IQR)]	39 (9–175) U/ml	44 (10–232) U/ml	0.562
Bilirubin level [Med (IQR)]	0.50 (0.30–13.25) mg/dl	0.6 (0.30–1.10) mg/dl	0.567
Preop. biliary drainage/stent	12 (33.33%)	22 (61.11%)	0.018
Comorbidities			
• Pulmonary disease (Asthma, COPD, etc.)	6 (16.67%)	6 (16.67%)	1.000
• Cardiac disease (coronary heart disease, myocardial infarction, etc.)	11 (30.56%)	10 (27.78%)	0.795
• Arterial hypertension	22 (61.11%)	21 (58.33%)	0.810
• Chronic pancreatitis	6 (16.67%)	8 (22.22%)	0.551
• Diabetes mellitus	11 (30.56%)	12 (33.33%)	0.800
• Nicotine abuse	15 (41.67%)	12 (33.33%)	0.633
• Alcohol abuse	7 (19.44%)	11 (30.56%)	0.523
Antiplatelet therapy	10 (27.78%)	5 (13.89%)	0.147
Anticoagulation	3 (8.33%)	2 (5.56%)	0.643
Surgical procedure (pp-Whipple/classical Whipple)	33/3 (91.67%/8.33%)	33/3 (91.67%/8.33%)	1.000
Pancreatic anastomosis (pancreatogastro-/pancreaticojejunostomy)	9/27 (25%/75%)	6/30 (16.67%/83.33%)	0.384
Operation time [Med (IQR)]	224 (192–288) min	222 (171–254) min	0.296
Estimated blood loss [Med (IQR)]	300 (225–310) ml	300 (200–350) ml	0.193

^*^Interval between first consultation and surgery

**Table 2:** Postoperative parameters

	**no prehabilitation**	**prehabilitation**	***p*-value**
Postoperative complications	24 (66.67%)	16 (44.44%)	0.053
Severe postoperative complications (Clavien-Dindo grade 3&4)	14 (38.89%)	6 (16.67%)	0.042
Mortality intrahosp. (Clavien-Dindo grade 5)	0	1 (2.78%)	0.314
Wound healing impairment	8 (22.22%)	4 (11.11%)	0.206
Pancreatic fistula	6 (16.67%)	5 (13.89%)	0.743
• Biochemical leak	3 (8.33%)	2 (5.56%)	
• Type B	1 (2.78%)	3 (8.33%)	
• Type C	2 (5.56%)	0	
Bile fistula	0	1 (2.78%)	0.314
Delayed gastric emptying	8 (22.22%)	12 (33.33%)	0.327
Haemorrhage	2 (5.56%)	0	0.151
Blood transfusion rate (no. of patients with ≥ 1 blood transfusion)	8 (22.22%)	2 (5.56%)	0.041
Cardiac complications (ACS, arrhythmia, etc.)	2 (5.56%)	4 (11.11%)	0.394
Pulmonary complications (pneumonia, pleural effusion, etc.)	10 (27.78%)	6 (16.67%)	0.257
Stay on ICU [Med (IQR)]	1 (1–1) d	1 (1–1) d	0.809
Hospital stay [Med (IQR)]	16 (14–25) d	13 (11–16) d	0.017
Readmission ≤ 30 d	5 (13.89%)	3 (8.33%)	0.453
Readmission ≤ 90 d	5 (13.89%)	4 (11.11%)	0.791

In another step, the influence of the basic demographic and intraoperative parameters as well as prehabilitation on postoperative complications was tested: In the univariate analysis, prehabilitation (*p* = 0.053), patients´ sex (*p* = 0.079), age (*p* = 0.055) and chronic pancreatitis (*p* = 0.024) were associated with a higher rate of postoperative complications. Multivariate analysis revealed prehabilitation as the factor with the strongest influence on postoperative complications (odds ratio 0.375, 95% CI [0.134–1.049]), *p* = 0.062) (Table 3).

**Table 3:** Univariate und multivariate analysis for postoperative complications

	**univariate analysis**			**multivariate analysis**		
	no postoperative complication (*n* = 32)	postoperative complication (*n* = 40)	*p*-value	odds ratio	95% CI	*p*-value
Prehabilitation	20 (62.50%)	16 (40.00%)	0.053	0.375	0.134–1.049	0.062
Age [Med (IQR)]	66 (59–73) y	71 (63–76) y	0.055	1.034	0.978–1.094	0.237
Sex (f/m)	8/24 (25.00%/75.00%)	18/22 (45.00%/55.00%)	0.079	2.626	0.880–7.835	0.083
BMI [Med (IQR)]	24 (20–25) kg/m^2^	24 (22–28) kg/m^2^	0.155			
ASA (ASA 2/ASA 3)	24/8 (75.00%/25.00%)	26/14 (65.00%/35.00%)				
Bilirubin level [Med (IQR)]	0.60 (0.30–1.13) mg/dl	0.55 (0.30–2.30) mg/dl	0.620			
Preop. biliary drainage/Stent	17 (53.13%)	17 (42.50%)	0.370			
Pulmonary disease	7 (21.88%)	5 (12.50%)	0.289			
Cardiac disease	9 (28.13%)	12 (30.00%)	0.862			
Arterial hypertension	17 (53.13%)	26 (65.00%)				
Chronic pancreatitis	10 (31.25%)	4 (10.00%)	0.024	0.376	0.088–1.617	0.189
Diabetes mellitus	8 (25.00%)	15 (37.50%)	0.258			
Nicotine abuse	16 (50.00%)	11 (27.50%)	0.128			
Alcohol abuse	10 (31.25%)	8 (20.00%)	0.223			
Antiplatelet therapy	4 (12.50%)	11 (227.50%)	0.119			
Anticoagulation	3 (9.38%)	2 (5.00%)	0.468			
Surgical procedure (pp-Whipple/classical Whipple)	30/2 (93.75%/6.25%)	36/4 (90.00%/10.00%)	0.567			
Pancreatic anastomosis (pancreatogastro-/pancreaticojejunostomy)	4/28 (12.50%/87.50%)	11/29 (27.50%/72.50%)	0.119			
Operation time [Med (IQR)]	225 (171–271) min	226 (181–2270) min	0.946			
Estimated blood loss [Med (IQR)]	300 (238–313) ml	300 (200–300) ml	0.200			

To address the possibility of residual confounding by age despite matching on sex, ASA class, and surgical procedure, an additional propensity score-based inverse probability of treatment weighting (IPTW) analysis was performed. In the primary IPTW model including age as a continuous variable, covariate balance after weighting was acceptable, with a maximum absolute standardized mean difference of 0.082. In the sensitivity analysis using age categories instead of continuous age, the maximum absolute standardized mean difference after weighting was 0.089. In the primary IPTW analysis, prehabilitation remained associated with lower odds of postoperative complications. For any postoperative complication, the weighted odds ratio was 0.425 (95% CI [0.154–1.169], p = 0.102). For severe postoperative complications Clavien-Dindo grade 3–4, the weighted odds ratio was 0.309 (95% CI [0.097–0.983], *p* = 0.051). Thus, the adjusted estimates consistently favored prehabilitation. The sensitivity analysis using age categories yielded similar results. The weighted odds ratio was 0.476 (95% CI [0.171–1.325], *p* = 0.160) for any postoperative complication and 0.311 (95% CI [0.097–0.994], *p* = 0.053) for Clavien-Dindo grade 3–4 complications. In additional multivariable logistic regression models, the direction of effect remained unchanged. The unadjusted odds ratio for any postoperative complication was 0.400, and the adjusted odds ratios were 0.407 when age was modeled continuously and 0.439 when age was modeled categorically, again indicating a consistent association in favor of prehabilitation without robust statistical significance.

The subgroup analysis of patients ≥ 80 years showed a significant reduction in the incidence of postoperative complications in the group of patients with prehabilitation (25% vs. 100%, difference −75%, *p* = 0.028). In addition, the postoperative hospital stay tended to be shorter, even in the very old patients, if they had undergone prehabilitation (14 vs. 26 days, difference −12 days, *p* = 0.057). There were no differences between the two groups regarding basic demographic or intraoperative parameters (Table 4).

**Table 4:** Subgroup analysis patients ≥ 80 years

	**no prehabilitation**	**prehabilitation**	***p*-value**
n	4	4	
Age [Med (IQR)]	83 (81–84) y	82 (81–83) y	0.486
Sex (w/m)	2/2 (50%/50%)	3/1 (75%/25%)	0.465
BMI [Med (IQR)]	25 (20–28) kg/m^2^	21 (20–24) kg/m^2^	0.486
Malignoma	4 (100%)	3 (75%)	0.285
Preoperative period* [Med (IQR)]	8 (6–16) d	16 (10–24) d	0.200
Pancreatic anastomosis (pancreatogastro-/pancreaticojejunostomy)	1/3 (25%/75%)	1/3 (25%/75%)	1.000
Operation time [Med (IQR)]	197 (133–307) min	197 (101–253) min	1.000
Estimated blood loss [Med (IQR)]	300 (225–305) ml	200 (113–363) ml	0.486
Postoperative complications	4 (100%)	1 (25%)	0.028
• Blood transfusion	1 (25%)	0	
• Atypical pneumonia + blood transfusion	1 (25%)	0	
• Postop. haemorrhage (pancreatogastrostomy)	1 (25%)	0	
• Pancreatic fistula Type C	1 (25%)	0	
• Perforation of the stomach	0	1 (25%)	
Stay on ICU [Med (IQR)]	2 (1–6) d	1 (1–5) d	0.686
Hospital stay [Med (IQR)]	26 (18–28) d	14 (12–22) d	0.057
Mortality intrahosp. (Clavien-Dindo grade 5)	0	0	1.000

^*^Interval between first consultation and surgery

## Discussion

At the authors´ institution, a high-volume center for pancreatic diseases certified by the German society for general and visceral surgery (DGAV), patients usually have to wait for about 2 weeks for surgery. Furthermore, it is well established that patients with pancreatic diseases requiring surgical treatment usually are in a reduced general and nutritional condition due to the catabolic nature of the disease and their advanced age as well. These factors contribute to the considerable high rate of postoperative complications after pancreatic surgery despite continuous improvements of the peri- and postoperative therapy (Gianotti et al. 2018). So, the prehabilitation program was introduced at the authors´ institution in order to prevent nutritional deterioration and improve patients´ fitness in anticipation of a potentially better postoperative outcome (Sliwinski et al. 2023; Nakajima et al. 2019).

One of the key questions is the optimal duration of prehabilitation, especially in patients with pancreatic diseases or pancreatic cancer, respectively. Most of the studies investigating the effect of prehabilitation on the postoperative outcome were performed during the neoadjuvant therapy in patients with oncologic diseases for which a preoperative therapy is recommended (Bausys et al. 2022; Molenaar et al. 2023). For patients with primary resectable pancreatic cancer or other malignancies around the pancreatic head, neoadjuvant therapy regimes are less established (Zhan et al. 2017; Gugenheim et al. 2022). So, a delay in the surgery in favor of patients´ prehabilitation inevitably involves the risk of tumor progression, which in the worst-case scenario may lead to unresectability (Sliwinski et al. 2023; Frountzas et al. 2022). Therefore, prehabilitation programs in patients without neoadjuvant strategies should last up to 3 weeks (Hanna et al. 2020). In the present study, the median period of prehabilitation were 16 days. Patients with less than seven days between initiation of prehabilitation and surgery were excluded from the analysis, which is in line with recommendations of Ausiana et al. (Ausania et al. 2019).

The results of the present study show that patients who underwent prehabilitation before pancreatoduodenectomy demonstrated a lower complication rate, although the difference narrowly missed statistical significance. The incidence of severe postoperative complications (Clavien-Dindo grade 3 and 4) was even significantly lower. Similarly, some previous studies showed a significant reduction of overall postoperative complications (Steffens et al. 2024), as well as several internal medical complications, especially pulmonary ones, in patients that underwent prehabilitation (Refaat et al. 2024; Kitahata et al. 2018). The limited number of participants in the present study, 36 patients in each group, might have contributed to the differences between the two groups being not significant with regard to the particular surgical or non-surgical complications. Another aspect that has to be considered when interpreting the postoperative outcome is the duration of prehabilitation. As previously described, the optimal duration of prehabilitation must be carefully balanced between the risk of tumor progression on the one hand and the time required to achieve the desired physical and physiological changes on the other. There are studies suggesting that up to eight weeks of preoperative respiratory physiotherapy may be necessary to improve patients´ pulmonary reserve postoperatively and reduce the risk of atelectasis (Refaat et al. 2024). In the present study, prehabilitation took 16 days on average. Adherence to the prehabilitation program is an additional aspect that is essential for the relevance and the success of the intervention (Refaat et al. 2024). In general, the compliance to prehabilitation as well as ERAS programs in pancreatic surgery seems to be low (Karunakaran et al. 2021). Indeed, a total of 49 patients (42.25%) were not eligible for the prehabilitation program and out of the remaining 67 patients (57.75%), a further 31 patients (26.72%) were lost for various reasons. Fatigue, taste aversion, comorbidities, insufficient support are only some barriers to prehabilitation mentioned by patients with diseases of the pancreas or even hepato-pancreatico-biliary cancer (Barnes et al. 2023). In this context, the special role of psychological support as one of the pillars of prehabilitation should be emphasized, as it can promote sense of purpose and acceptance of the program through moral support and patient motivation (Grimmett et al. 2022). In contrast, only about one third of patients (36 patients, 31.03%) followed the entire program. Considering the above, it may be assumed, that highly motivated and physically fitter patients were overrepresented in this group. This potential selection bias should be considered when interpreting the results and may limit the generalizability of our findings. In the present study, the focus was placed on nutritional therapy, breathing exercises and endurance as well as muscle strengthening training. However, a study nurse contacted patients regularly during the prehabilitation in order to increase their motivation and thereby improve the adherence to the program.

After the surgery, patients spent an average of one day in intensive care unit, regardless of whether they underwent prehabilitation or not. However, after prehabilitation, patients´ median hospital stay was three days shorter (13 vs. 16 d, *p* = 0.017). These results are consistent with the studies of Kitahata et al. and Nakajima et al., who also found a significant reduction in the length of hospital stay for patients with prehabilitation after pancreatoduodenectomy or hepato-pancreatico-biliary surgery (Nakajima et al. 2019; Kitahata et al. 2018).

The subgroup analysis of patients ≥ 80 years confirms the aforementioned results. There was a significant difference in the incidence of postoperative complications between the two groups: All patients in the control group versus one patient after prehabilitation suffered one or more postoperative complications (*p* = 0.028). In particular, pulmonary complications or the need for postoperative blood transfusion did not occur in the prehabilitation group. Previous studies confirm our results that prehabilitation can have a beneficial effect on these postoperative complications in particular (Banasiewicz et al. 2023; Sobczyk et al. 2024).

So far, data about the economic potential of prehabilitation are scarce. Nevertheless, it can be assumed that the reduction in postoperative length of stay and complication rates not only save hospital resources but is also associated with a relevant reduction in treatment costs. Furthermore, the reduced use of intensive care resources and shorter hospital stays can increase hospital bed turnover rate, thereby enabling other patients to access their oncological surgery earlier (Sliwinski et al. 2023; Nakajima et al. 2019).

This study has several limitations that should be acknowledged. First, the study design was a retrospective matched-pair analysis comparing patients treated before and after the implementation of a structured prehabilitation program at a single institution. Although matching was performed for sex, ASA class, and type of surgical procedure (pylorus preserving versus classical Whipple procedure), other potentially relevant confounders such as age, preoperative biliary drainage, pancreatic texture and duct size, and detailed nutritional risk parameters (e.g., NRS) were not included in the matching algorithm. While baseline characteristics were largely comparable between groups and age did not significantly differ, residual confounding cannot be excluded, particularly because chronological age may still reflect relevant differences in physiological reserve even among patients with the same ASA classification. Since formal frailty or performance scores were not systematically available in the historical control cohort, an additional propensity score-based IPTW analysis was performed that explicitly incorporated age together with other relevant preoperative covariates. After weighting, covariate balance was acceptable, and the adjusted analyses consistently showed lower odds of postoperative complications in patients who underwent prehabilitation. Even if these estimates mostly remained just above the conventional threshold for statistical significance, they indicate that the observed effect is directionally consistent but should be interpreted with caution. Importantly, the age-adjusted analyses do not overturn the main clinical message of the study. In particular, they suggest that the beneficial association between prehabilitation and postoperative outcome is not solely explained by age imbalance, while at the same time underlining the limited statistical power of this cohort. The strongest adjusted signal was observed for severe complications, which is clinically plausible and consistent with the idea that prehabilitation may improve physiological reserve rather than eliminate all postoperative morbidity.

Second, the sample size was limited, with 36 patients in each group. This restricts statistical power and may explain why some clinically relevant differences did not reach statistical significance, as mentioned before. Larger multicenter studies are required to confirm the observed associations. Third, adherence to the prehabilitation program was limited. Only approximately one third of initially screened patients completed the entire program and were included in the final analysis. This is in the same line of evidence as observed by other groups who also found a high number of patients not running the prehabilitation program until surgery (Berkel et al. 2022). However, this introduces the possibility of selection bias, as patients who successfully completed prehabilitation may have been more motivated, functionally fitter, or socially better supported than those who did not. Consequently, the generalizability of the findings may be restricted. Fourth, supervision of the intervention was home-based and primarily monitored via logbooks and telephone follow-up. Objective performance measures or standardized assessments of functional improvement were not systematically recorded. Therefore, the exact magnitude of physiological improvement attributable to prehabilitation cannot be quantified. Finally, the single-center nature of the study may limit external validity. Institutional standards, surgical expertise, and perioperative care pathways may differ across centers, potentially influencing outcomes.

Future investigations should ideally employ prospective randomized designs or robust propensity score matching incorporating frailty indices, performance status or objective functional capacity testing, and detailed nutritional parameters. Such approaches would further reduce confounding and allow more precise estimation of the true effect of prehabilitation in patients undergoing pancreatoduodenectomy.

## Conclusions

In summary, the present study suggests that prehabilitation may improve postoperative outcomes after pancreatoduodenectomy, with a consistent trend toward fewer overall and severe complications and a shorter postoperative hospital stay. Particularly in older patients, prehabilitation significantly improved outcomes, suggesting its potential as an effective strategy for enhancing physiological resilience and optimizing recovery after major pancreatic surgeries.

## Data Availability

The datasets used and/or analysed during the current study are available from the corresponding author on reasonable request.
